# Characteristics of a palliative care consultation service with a focus on pain in a German university hospital

**DOI:** 10.1186/1472-684X-13-45

**Published:** 2014-09-24

**Authors:** Joachim Erlenwein, Almut Geyer, Julia Schlink, Frank Petzke, Friedemann Nauck, Bernd Alt-Epping

**Affiliations:** 1 Department of Pain Medicine, Center for Anesthesiology, Emergency and Intensive Care Medicine, University Medical Center Göttingen, Georg-August-University of Göttingen, Robert-Koch-Str. 40, 37075 Göttingen, Germany; 2 Department of Palliative Medicine, Center for Anesthesiology, Emergency and Intensive Care Medicine, University Medical Center Göttingen, Georg-August-University of Göttingen, Robert-Koch-Str. 40, 37075 Göttingen, Germany

## Abstract

**Background:**

A minority of patients with incurable and advanced disease receive specialised palliative care. Specialised palliative care services that complement the care of difficult and complex cases ought to be integrated with services that deliver general care for most patients. A typical setting in which this integrative concept takes place is the hospital setting, where patients suffering from incurable and advanced disease are treated in many different departments.

The aim of the study is to investigate the profile and spectrum of a palliative care consultation service (PCCS) at a German university hospital with special reference to pain therapy.

**Methods:**

We retrospectively analysed the PCCS documentation of three years.

**Results:**

Most patients were referred from non-surgical departments, 72% were inpatients, and 28% were outpatients. 98% of the patients suffered from cancer. Counselling in pain therapy was one of the key aspects of the consultation: For 76% of all consulted patients, modifications of the analgesic regimen were recommended, which involved opioids in 96%. Recommendations on breakthrough-pain medication were made for 70% of the patients; this was an opioid in most cases (68%). The most commonly used opioid was morphine. For 17% of the patients, additional diagnostic procedures were recommended. Besides pain management palliative care consultation implied a wide range of recommendations and services: In addition to organising home care infrastructure, palliative care services supported patients and their families in understanding the life-limiting diseases. They also coordinated physical therapy and social and legal advice.

**Conclusion:**

This survey clearly shows that for a consultation service to support patients with incurable or advanced disease, a multi-disciplinary approach is necessary to meet the complex requirements of a needs-adapted palliative care in inpatient or outpatient settings. Timely integration of palliative expertise may support symptom control and may give the required advice to patients, their carers, and their families.

## Background

It is recognized increasingly more often that comprehensive care of cancer patients includes not only life-saving or life-prolonging anticancer treatment, but also palliative care, which focuses on symptoms and other physical, psycho-social, or spiritual needs. Palliative and hospice care are thus evolving into an established feature in the overall medical system. Still, a minority of patients will actually receive specialised palliative care services during the course of their disease [1]. Thus, specialised palliative care services that complement the care of difficult and complex cases ought to be integrated with services that deliver general care for most patients [2,3]. A typical setting in which this integrative concept takes place is the hospital setting, where patients suffering from incurable and advanced disease are treated in many different departments. The need for integrative care structures is also supported in the literature, where reports indicate that there are still shortcomings in the quality of care these patients and their relatives receive, including inadequate symptom and pain control, and non-beneficial and unnecessary interventions [4-6]. Furthermore, despite growing efforts of improving pain management in recent years, there is still a considerable gap between potential and actual achievements in pain control in these patients [5,7-10].

Prevalence of pain in cancer patients varies depending on the different types and stages of cancer. Even at early stages 33% of the patients suffer pain, which increases to 59% during anticancer treatment and to up to 64% for patients with metastatic or advanced stages of disease. Overall, more than 80% of cancer patients experience pain caused by their disease, its treatment, or treatment-related complications [11]. Between 31% to 45% of these patients describe their pain at least as moderate to severe [12].

Palliative care consultation services (PCCS) seem to be an adequate approach to improve the care of patients with complex palliative care needs in hospitals and, depending on structural preconditions, even in outpatient care. Previous studies have shown that PCCS improve the quality of life, quality of care, and satisfaction of patients with advanced illness and their families [2,13,14].

Even if structures and involvement of PCCS have been examined in other studies, little is known about the working profile of these services in German hospitals [13,15-17]. Precise information about the concept and mode of action of these services might be necessary to improve hospital infrastructure and care pathways. Furthermore, structural insight might be essential for establishing standards of care and for providing a framework for the financial requirements of these services.

The purpose of this study is therefore to determine the characteristics and needs of the patients that are being seen by PCCS, the reasons why PCCS became involved, and an analysis of the recommendations made by the PCCS. The surveyed PCCS is provided by the Department of Palliative Medicine of Göttingen University Hospital, which provides counselling in cancer-related pain syndromes and all other matters of physical, psycho-social, or spiritual palliative care.

## Methods

We retrospectively and anonymously analysed all patient contacts of the PCCS at Göttingen University Hospital between January 2008 and December 2010 with a standardized protocol. The analysis included only contacts with a palliative care physician, contacts with nurses were excluded. We included all patients that were referred from departments other than the Department of Palliative Medicine. Most patients were seen as inpatients, but outpatient referral was possible (for instance, referrals from the outpatient oncology clinics that are structurally linked to the university hospital). This survey was approved by the local ethics authorities (Ethics Committee of the University Medical Center Göttingen, No. 6/5/11). Theses routine and anonymous data-analysis was performed without an additional informed consent, according to the local ethical standard.

### Analysis of the consultation

The analysis included the actual request and problem as described by the referring department, information on the medical history as documented on the request form, an assessment of the documented problems, the recommended medical treatment (e.g. substances and preparations), and further recommendations. We focused here on pain and the recommended pain therapy. For comparison, opioid doses were expressed as oral morphine equivalent (conversion factor to morphine: oxycodone 0.75, hydromorphone 0.13, piritramide 1.5, fentanyl 0.01, tilidine/tramadol 10, buprenorphine 0.03, intravenous vs. oral morphine 3:1). Previous medications were not explicitly recorded, but the extent to which opioid doses were adapted was compared with the medication prescribed before the PCCS intervention (based on the morphine-equivalent), no change, a reduction, or an increase in pain medication. Data on length of stay of inpatients were taken from the electronic clinical information system.

### Data analysis

We anonymised the data, and the analysis was performed primarily descriptively. Percentages were related to the total number of surveyed patients unless stated otherwise. We statistically analysed the differences between patients of surgical and non-surgical departments using the chi-square test according to Pearson. Continuous variables were compared with a t-test for dependent samples. The level of significance was set to p <0.05. The p-value was not adjusted for multiple testing for groups because of the explorative character of the study. The three pediatric patients were included in the descriptive results, but their data were not used for group comparisons because of the small-number statistics.

## Results

### Patients

273 patients during the time of January 2008 to December 2010 were included (53% male, n = 144 and 47% female, n = 129, from 3 to 89 years old, average age 62 ± 13,6 years, median 64 years). Table 1 shows the departments that referred the patients. Nearly half of all patients were referred from the departments of internal medicine (45%). 72% were inpatients, 28% were outpatients. Overall, 950 physician contacts were documented (mean 3,5 ± 3,5, median 2, first to third quartile 1–5 contacts/patient (min 1, max 25 contacts).

**Table 1:** Referring departments

**Patients’ affiliations**					
**Surgical departments**	(n = 98)	36%	Operative intensive care	(n = 4)	2%
			General and visceral surgery	(n = 34)	13%
			Orthopedic and trauma surgery	(n = 6)	2%
			Neurosurgery	(n = 8)	3%
			Thorax, heart and vascular surgery	(n = 1)	<1%
			Urology	(n = 13)	5%
			Gynecology	(n = 26)	10%
			ENT	(n = 4)	2%
			Oral and maxillofacial surgery	(n = 2)	1%
**Non-surgical departments**	(n = 172)	63%	Internal medicine	(n = 124)	45%
			Neurology	(n = 13)	5%
			Radiotherapy	(n = 29)	11%
			Radiology	(n = 1)	<1%
			Psychiatry	(n = 1)	<1%
			Dermatology	(n = 4)	2%
**Pediatric departments**	(n = 3)	1%			

The average length of hospitalization for inpatients was 14,4 ± 16,8 days (median 11 days, min. 0 and max. 139 days); the time from admission to hospital to the first contact with the palliative care consultation service was 7,7 ± 13,6 days (median 7 days, min. 1, max. 138 days) for inpatients without significant difference between patients from the various departments.

Almost all PCCS patients suffered from cancer (98%, n = 267), the most common cancer entity was lung cancer, followed by colorectal cancer (Table 2). Three patients suffered from neurodegenerative disease, two had chronic organ failure, and two patients "other" underlying disorders. The primary reason for hospitalization was surgery for 9% of the patients, uncontrolled pain for 9%, chemotherapy for 19%, radiation therapy for 14%, diagnostic workup for 8%, and a deteriorating health condition for 36% (e.g. related to general systemic tumor progression, disease-related or treatment-related complications).

**Table 2:** Cancer entities

**Cancer entities**	**n**	**%**
Lung	45	16
Colorectal	32	12
Head and neck	29	11
Urological	29	11
Others	28	10
Breast	27	10
Hematological	21	8
Pancreas	21	8
Gynecological	18	7
Esophagus	8	3
Unknown primary site (CUP)	6	2
Liver	5	2

One explicit reason for a palliative care consultation (multiple options) was pain in 64% of all PCCS patients. In 5% of all consultations, the side effects of pain treatment were reasons for a palliative care consultation. The dominating type of pain was somatic pain (e.g. bone/muscle, 49%), followed by visceral (21%), neuropathic (15%) and mixed types of pain (15%). Underlying causes included chronic pain syndromes related to cancer (67%), acute pain syndromes presumably related to cancer in the presence of pre-existing chronic cancer pain (22%), acute post-operative pain in addition to chronic cancer pain syndromes (4%), both cancer pain and chronic (non-cancer) pain (4%), acute non-surgical pain syndromes (2%), and chronic pain (1%).

In 35% of cases, the PCCS was involved in order to induce a transfer of the patient to the palliative care unit (15%) or to establish contact to the palliative home-care team (20%). In 46% of cases, an overall assessment of palliative care needs or a general consultation was requested. In 5% of cases, no specific request or aim of the consultation could be identified on the consultation form. In 17% of the requests, the PCCS was explicitly asked for a follow-up visit.

### Therapeutic recommendations

For 76% (n = 208) of all PCCS-patients, modifications of the analgesic regimen were recommended. Of these consultations concerning analgesics in 96% (n = 197) opioids were used. Comparing the patients’ previous opioid dose with the recommended dose (as calculated via the opioid-equivalent dose), it was recommended to increase the opioid dose in 32% of patients, to limit the dose in 1%, and to reduce the dose in 6% of all consultations addressing analgesics. In 15% of patients, an opioid rotation was recommended.

70% (n = 186) of all patients were recommended to receive an on-demand/breakthrough-pain analgesic, which was an opioid in most cases (97% of all on-demand medications). The average equivalent daily dose of morphine of the recommended baseline opioid was 107.3 ± 90.8 mg. The most often used opioid was morphine (Table 3). Table 4 shows the pattern of recommendations of different opioids and the respective galenic preparation.

**Table 3:** Recommended opioids and their galenic preparation (in% of all recommended opioids)

	**Overall opioids**	**Prolonged-release preparations**	**Immediate-release preparations**
Morphine	87%	70%	25%
Hydromorphone	33%	18%	20%
Fentanyl	31%	37%	1%
Oxycodone	14%	5%	11%
Tramadol	10%	5%	6%
Tilidine	1%	1%	1%
Levomethadone	1%	1%	-
Buprenorphine	1%	1%	-
*multiple options possible*			

**Table 4:** Comparison between patients in surgical and non-surgical departments

	**Surgical**	**Non-surgical**	**Statistics**
	**(n = 98)**	**(n = 172)**	
Contacts with the PCCS [n]	4,4 ± 4,1	2.9 ± 3,1	p = 0,004, T = 3,176
Length of hospitalization (only inpatients) [days]	13,8 ± 14,5	14,9 ± 18,2	n.s., T = -0,527
Days from admission to hospital to the first contact with the PCCS (only inpatients) [days]	6,7 ± 8,7 min 0, max 36	8,4 ± 15,7 min 0, max 138	n.s., T = -1,023
Gender [men/women]	58%/42%	45%/55%	p = 0,03, Chi^2^ = 4,017
Average equivalent daily dose of morphine [mg]	115 ± 93	104 ± 90	n.s., T = 0,738
An additional rescue analgesic [%]	72	69	n.s. Chi^2^ = 5,125
Opioid dose adaption [%]	32	40	n.s. Chi^2^ = 1,495
Opioid rotation [%]	9	18	n.s., Chi^2^ = 2,694

The oral route was the most often recommended route of application for opioid drugs (86%; *multiple options*). In 71% of the patients with opioids, other routes were additionally recommended (transdermal 17%, intravenous 13%, subcutaneous 6%, sublingual 2%, via gastrostomy tube 1%). A dedicated invasive technique, such as patient-controlled analgesia or epidural analgesia, was recommended for 2% of all patients.

54% of all patients received at least one non-opioid drug. The most frequently used non-opioid drug was dipyrone (metamizol) (77% of all non-opioid administrations, *multiple options*), followed by ibuprofen (13%), diclofenac (5%), paracetamol (4%), and selective COX-2-inhibitors (1%).

43% of all patients received co-analgesics. 28% of these received pregabalin or gabapentin, 1% carbamazepine or lamotrigine, 23% tricyclic antidepressants, 5% other antidepressants, and 43% received steroids.

Adjuvant medication was recommended by the PCCS for 59% of all patients: for 36% of these patients anti-emetics were recommended, laxatives for 21%, antacids for 20%, and sedatives for 15%. In 7% of all patients, physiotherapy was recommended, for another 5% psychological co-treatment was suggested. In addition, the PCCS recommended that 17% of all patients should obtain an additional diagnostic work-up. A re-consultation was advised for 82% of the patients.

### PCCS recommendations for care planning

The involvement of further specialist palliative care structures was suggested by the PCCS for 89% of all patients. Specialised palliative home-care was recommended for 50% of these patients, while admittance to the palliative care unit was recommended for 24% of the patients. One patient was recommended to receive hospice care. The remaining 23% of the patients were recommended to become involved an outpatient hospice service. Only 11% patients received no recommendation for further special palliative care. During the course of PCCS contacts or after discharge from the hospital, almost 87% of all PCCS patients were referred to palliative home care or to the palliative care unit.

In addition to these recommendations, the PCCS explored further care needs (62%) and initiated different modes of support options for 26% of the patients. These additional recommendations included applications for professional nursing-home care for 14%, assisting devices for 15%, suggesting the implementation of a health care power of attorney for 1%, and completing an advance directive for 2% of the PCCS patients.

Other PCCS tasks were to explain the different options for comprehensive support, such as the different levels of specialised palliative care (65%). General counselling about the disease, the current situation, and prognosis took place by addressing the patient by himself (21%), solely addressing the family members (6%), or addressing both patient and family (35%).

### Differences between surgical and non-surgical departments

Interestingly, surgical patients had more frequent contact with the PCCS (see also Table 4). The time after which a patient was referred to the PCCS after their admission to hospital was similar for surgical and non-surgical departments (only inpatients). The reasons for involving the PCCS were similar as well. The frequency of pain as an indication for PCCS involvement was comparable (n.s., Chi^2^ = 0.001), as was the frequency of adverse events of pharmacological pain treatment (n.s., Chi^2^ = 1.038) or pain type (n.s., Chi^2^ = 5.863). As expected, more acute and post-operative pain syndromes were seen in surgical departments (p = 0.027, Chi^2^ = 12.631). This had no consequences for the frequency of analgesic recommendations, however (n.s, Chi^2^ = 0.031) (see also Table 4).

No significant differences between surgical and non-surgical departments were detected in the recommendations for physiotherapy (n.s., Chi^2^ = 3.094), special analgesic techniques (Chi^2^ = 1.771), further diagnostics (n.s., Chi^2^ = 1.551), social support (n.s., Chi^2^ = 11.352), psychological support (n.s., Chi^2^ = 2.529), counselling of family members (n.s., Chi^2^ = 2.235), or organisational efforts (n.s., Chi^2^ = 1.500). Similar numbers of patients were recommended to receive the benefit of other specialised palliative care structures involved (n.s., Chi^2^ = 0.592).

## Discussion

The PCCS addresses a wide range of complex medical and psycho-social problems [2]. In addition to providing support in organisational tasks and developing individual concepts for comprehensive patient care and treatment, consultations on pain management are a major priority for about two thirds of the situations where the PCCS is involved. Most patients were treated with opioid medications before; consultations therefore mainly addressed questions of dose escalation or adaptation, a suitable choice of opioid, and management of breakthrough pain. The frequent recommendations for improving on-demand/breakthrough analgesics clearly indicate the importance of this topic in teaching and concepts of care.

Pain syndromes were not related to cancer alone. Both chronic non-cancer and acute post-interventional pain were among the concerns brought to the PCCS. Few patients suffered from multiple causes of pain (e.g. acute and/or chronic; cancer and/or non-cancer related), which resulted in various possible acute, chronic, or cancer pain phenomena. Such multiple causes of pain were also found in other settings, for instance, in patients seen by a specialised pain consultation service of a university hospital [18,19]. Pre-existing chronic pain is a risk factor for both increased acute and chronic post-operative pain [20,21]. Whether similar relationships influence pain management in cancer patients or patients at the end of life is an important question with potential therapeutic implications. This needs to be investigated in further studies.

It is difficult to compare these results with other studies on PCCS because medical care, hospital systems, and palliative care structures differ. In addition, the term "consultation service" is not used homogeneously in the context of the palliative care [2,16,17,22,23]. The distribution of diagnosed cancer entities and the high relevance of cancer pain we found is similar to what was reported in other studies [8,23,24], as were the recommendations that were made. However, we are unable to specify from our data whether and how the recommendations were implemented [25].

Considering the fact that PCCS patients suffered from complex problems, and the broad spectrum of relevant recommendations and actions observed following the consultation, it is interesting to see that it took on average about 7–8 days in hospital before contact to the PCCS was sought. This shows that a PCCS referral may be preceded by a period of consideration and observation by both the treating oncological discipline and/or the patient, and that referral is not established as a clinical routine. This also suggests that cooperation between departments and the PCCS can be further improved. Information on the PCCS needs to be readily available and accessible for staff and patients throughout the hospital [7]. Based on our results, we have to assume that the aim to fully integrate palliative care expertise still poses challenges not only in the context of different health care sectors, but also within a hospital. It remains unclear whether this is related to the organisational culture of large tertiary hospitals, where work processes are organised mostly within distinct departments, a lack of knowledge about the options and intentions of specialised palliative care inside the hospital, or a general attitude of non-palliative care departments that impedes a simultaneous approach of cause-directed and palliative treatment options.

In a proportion of patients (approximately 17%) further diagnostic investigations for palliative care reasons were recommended. These recommendations demonstrate that palliative care is not just "symptom palliation", but also contributes to the overall medical management. The broad range of recommendations is in line with this viewpoint because the recommendations included approaches to physical, nursing-related, and psycho-social problems.

A number of limitations of our study need be mentioned. This study was based on data from a university hospital and did neither intend to assess symptom prevalence or intensity, nor was the actual implementation of the recommendations and their effects evaluated. All patients seen by the PCCS were included, without classification of disease severity, other symptoms, or side effects. A statement on the influence of PCCS on the resulting overall quality of care or the severity of the palliative status is thus not possible here.

Another question is the financial setup of such services in German hospitals. The discussion about the cost effectiveness of care and support of other departments provided by an acute pain service is well-known [26,27]. Several studies have shown that specialised palliative care teams may help to reduce hospital costs. The German reimbursement system, which uses diagnosis-related groups (DRG), allows recompensation of palliative care on non-palliative care units under certain preconditions [2,28-30]. If no internal redistribution between the involved departments takes place, it will be difficult to obtain funding for the department that provides the PCCS.

Our results show that the PCCS has an important role in facilitating consecutive inpatient or outpatient guidance and acquisition of patients. A future challenge will be to reduce departmental and sectorial boundaries to achieve an integrated care for the patients who suffer from complex needs.

Finally, this analysis of a PCCS supports two important statements about palliative care. A multi-professional approach that requires a team with the respective qualifications is necessary to meet the complex needs in the end-of-life care and to adequately support the patients, their families, and the referring departments. Timely and possibly early integration of palliative expertise may lead to improved symptom control and advice to patients, their carers, and their families.

## Conclusion

Our survey clearly showed that to support patients with incurable or advanced disease by a consultation service, a multi-disciplinary approach is necessary. Only this service can properly meet the complex requirements of a needs-adapted palliative care in inpatient or outpatient settings. Pain is one of the common topics, in some cases even complex pain-problems that include acute or chronic pain, but typically, these patients have several other needs in addition to this, including psycho-social or social work. Especially the planning of further care provision, including discharge planning, is an important need.

Timely integration of palliative expertise may support symptom control and may give the required advice to patients, their carers, and their families. Our study reflects clinical practice; this may limit it somewhat because we did not assess symptom prevalence or intensity or evaluated the implementation of the recommendations of the PCCS and their effects.

## Competing interests

The authors declare that they have no competing interests.

## Authors’ contributions

JE, AG, and JS collected the data; JE, FP, BA-E, and FN conceived the study and participated in its design and coordination; JE, FP, and BA-E performed the statistical analysis; JE, AG, FP, and BA-E drafted the manuscript. All authors read and approved the final manuscript.

## Pre-publication history

The pre-publication history for this paper can be accessed here:

http://www.biomedcentral.com/1472-684X/13/45/prepub
